# Polysaccharide κ-Carrageenan as Doping Agent in Conductive Coatings for Electrochemical Controlled Release of Dexamethasone at Therapeutic Doses

**DOI:** 10.3390/molecules25092139

**Published:** 2020-05-03

**Authors:** Karla Ramírez Sánchez, Aura Ledezma-Espinoza, Andrés Sánchez-Kopper, Esteban Avendaño-Soto, Mónica Prado, Ricardo Starbird Perez

**Affiliations:** 1 Centro de Investigación y de Servicios Químicos y Microbiológicos (CEQIATEC), School of Chemistry, Instituto Tecnológico de Costa Rica, 159-7050 Cartago, Costa Rica; aledezma@itcr.ac.cr (A.L.-E.); ansanchez@itcr.ac.cr (A.S.-K.); 2 Centro de Investigación en Enfermedades Tropicales (CIET), Faculty of Microbiology, Universidad de Costa Rica, 11501-2060 San José, Costa Rica; monica.pradoporras@ucr.ac.cr; 3 Centro de Investigación en Ciencia e Ingeniería de Materiales (CICIMA), Universidad de Costa Rica, 11501-2060 San José, Costa Rica; esteban.avendanosoto@ucr.ac.cr; 4 School of Physics, Universidad de Costa Rica, 11501-2060 San José, Costa Rica

**Keywords:** polysaccharide, κ-carrageenan, dexamethasone, electrochemical active deliver system, doping agent, charged molecule, conductive polymers

## Abstract

Smart conductive materials are developed in regenerative medicine to promote a controlled release profile of charged bioactive agents in the vicinity of implants. The incorporation and the active electrochemical release of the charged compounds into the organic conductive coating is achieved due to its intrinsic electrical properties. The anti-inflammatory drug dexamethasone was added during the polymerization, and its subsequent release at therapeutic doses was reached by electrical stimulation. In this work, a Poly (3,4-ethylenedioxythiophene): κ-carrageenan: dexamethasone film was prepared, and κ-carrageenan was incorporated to keep the electrochemical and physical stability of the electroactive matrix. The presence of κ-carrageenan and dexamethasone in the conductive film was confirmed by µ-Raman spectroscopy and their effect in the topographic was studied using profilometry. The dexamethasone release process was evaluated by cyclic voltammetry and High-Resolution mass spectrometry. In conclusion, κ-carrageenan as a doping agent improves the electrical properties of the conductive layer allowing the release of dexamethasone at therapeutic levels by electrochemical stimulation, providing a stable system to be used in organic bioelectronics systems.

## 1. Introduction

Conductive polymers are a new generation of smart materials extensively used in organic bioelectronics, mostly in the development of neural implants, biosensors, and active controlled release systems [1,2,3,4]. Poly (3,4-ethylenedioxythiophene) (PEDOT) is a conductive polymer synthetized from 3,4-ethylenedioxythiophene (EDOT), used as a coating in diverse types of sensors due to its biocompatibility, conductivity, processing versatility, and stability [5,6]. Moreover, PEDOT is reported as a promising material for the immobilization of enzymes and other biologically active molecules [2,7,8]. The incorporation of charged molecules into the PEDOT backbone is described through an electrostatic mechanism due to the formation of charge carriers and the doping process during the electropolymerization process [9]. The subsequent release of the charged compounds was reported to be dependent on the polymer thickness and charge applied during the electrochemical stimulus [10,11,12,13].

Diverse implants and scaffolds are developed in regenerative medicine to serve as extracellular matrices for cell colonization [14,15,16]. Many of them are loaded with bioactive agents to improve the therapeutic efficacy and safety of the drugs, playing important roles in treatment of several chronic diseases, damaged tissues, and providing a potential stimulation of different types of cells [17,18,19].

Although diverse engineering groups established different types of implants for a broad range of applications, those implants can elicit body responses involving inflammatory processes, which may result in the formation of glial scars due to neural devices specifically [12,13,20,21]. One strategy to avoid immune responses consists of releasing an anti-inflammatory biomolecule (i.e., dexamethasone) in the vicinity of the implant [11,13,22,23]. Dexamethasone (Dx) is a synthetic glucocorticoid that reduces inflammation in the central nervous system, acting through glucocorticoid receptors found in most neurons and glial cells. Due to being locally delivered, the specificity and efficiency of dexamethasone means that only small amounts of the drug are required [13,22,24,25,26,27,28].

κ-Carrageenan (κC) is a sulfonated polysaccharide recently used in aqueous micellar dispersions for the polymerization of EDOT, since it provides an appropriate environment for the monomer dispersion while acting as a doping agent in the conductive layer [29,30,31]. According to the previous work, the electrochemical properties of PEDOT are retained when κC is used as a doping agent [29,30], avoiding a potential delamination during the reduction-oxidation process needed during the active delivering process. Biocompatibility of PEDOT:κC composite has been demonstrated in previous studies [2,29].

In this work, we induce the loading of dexamethasone phosphate during the deposition of the electroactive composite onto a bare gold electrode by changing the amount of drug in the dispersion prior the polymerization. κC was incorporated to maintain the electrochemical stability and biocompatibility of the PEDOT matrix and the subsequent drug release using electrical stimulation. The presence of κC and Dx inside the conductive film was confirmed by µ-Raman spectroscopy and their effect in the topography was studied using profilometry. Dexamethasone release was evaluated by cyclic voltammetry and High-Resolution (HR) mass spectrometry. Therapeutic doses of dexamethasone were achieved during the electrical stimulation of the bioelectronic device.

## 2. Results and Discussion

### 2.1. Evaluation of the Stability and Size of the Dispersion Systems

The dispersions used to electrodeposit the monomer and the Dx on the electrode were evaluated by their ζ-potential values and particle size distribution in order to determine its stability in aqueous medium. ζ-potential data was obtained for the six prepared dispersions, and they are shown in Table 1. It is possible to observe that EDOT:κC:Dx has an appropriate stability (−48.70 mV), which is dominated for the κC micellar system (−43.30 mV). Values of ζ-potential over −30 mV are considered stable assuming that an electrostatic charge is the main stabilization mechanism and the colloidal system is in the range of hundreds [32,33]. The anionic nature of the κC and Dx avoids aggregation due to the negative values obtained in the ζ-potential analysis, which are comparable with previously reported results for these molecules [30,34,35]. A stable dispersion prevents aggregation or deposition of the particles that carried the monomer during the electrochemical deposition. Additionally, the stable system may allow a homogeneous dispersion of κC and dexamethasone in the electrodeposited film as seen by Raman spectroscopy.

**Figure 1:**
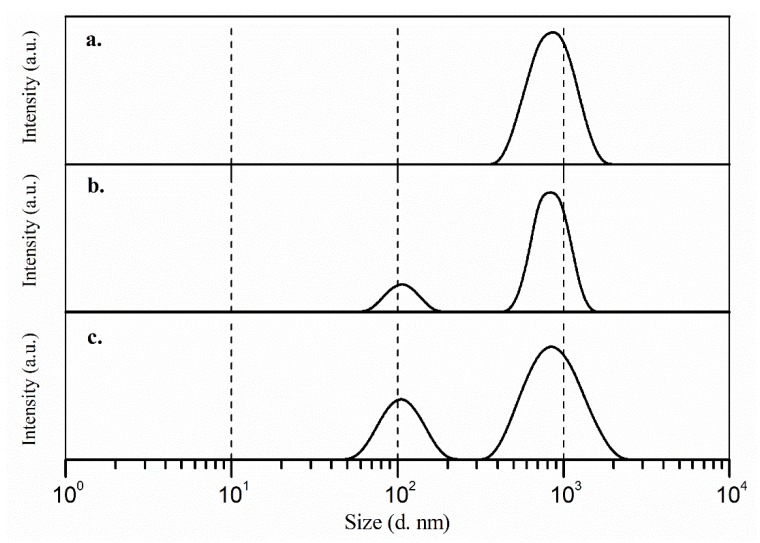
Size distribution (d. nm) of (**a**) κC; (**b**) κC:Dx; and (**c**) EDOT:κC:Dx dispersions, measured by dynamic light scattering (DLS) method.

Particle size measurements of the main three dispersions were performed to determine the dimension of their aggregates after the sonication process. Figure 1a shows the size distribution for the κC 0.2% *w*/*v* solution, it is possible to observe a single population for the surfactant. Some authors have reported previously that κC solutions are polydisperse (two or more populations), because it increases the gel behavior due to its polysaccharide nature [36,37]. Nevertheless, they emphasized that the main signal for the κC aggregates has an average size in the range of 800 to 1000 nm [37], which agrees with our results. The intensive sonication process before the measures and the low concentration of κC used in the analysis may explain why only one population were observed in the κC size distribution, similar to a previous report [30].

On the other hand, once the Dx was added to the dispersion, a polydisperse behavior was found in the κC:Dx system and two populations were detected (Figure 1b,c). Dexamethasone solutions are characterized by a single population with a particle size average of 100 nm [38] and was consistent with our results. Eventually, it is possible to observe that the stability of the system has remained when the monomer was added (Figure 1c). The stability of the dispersions depends mainly on the used surfactant and it has an important influence in the physical and electrochemical properties of the electrodeposited films [39].

### 2.2. Analysis of the Topography and Composition of PEDOT:κC:Dx Coating by µ-Raman Spectroscopy and Profilometry Methods

**Figure 2:**
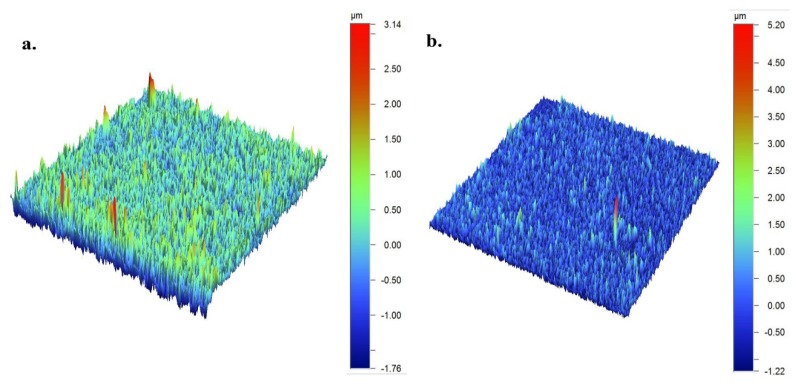
Profilometry images obtained for PEDOT:κC:Dx films (**a**) before and (**b**) after 160 cycles of cyclic voltammetry in a 0.10 M ammonium acetate solution.

The PEDOT:κC:Dx composite was obtained from a EDOT:κC:Dx dispersion by electrochemical deposition under galvanostatic conditions (Figure S1), as it was established in a previous work [2,30]. Then, the topography of the PEDOT:κC:Dx coating was characterized before (S_a_: 0.270 ± 0.005 µm, surface area: 1361 mm^2^, negative volume 0.1562 mm^3^, and volume 1.695 mm^3^) and after (S_a_: 0.250 ± 0.005 µm, surface area: 1337 mm^2^, negative volume 0.1707 mm^3^, and volume 1.690 mm^3^) releasing the Dx from the conductive coating. The roughness data of both surfaces did not show significant differences between them (see Figure 2a,b). The volume ratio between peaks and valleys describes the symmetry in the surface topography. A negative value is indicative of more distinct valleys and positive of more distinct peaks about the average plane. Our samples were dominated by peaks and low negative volume (around ten times) and those values are consistent with a previous report for PEDOT:κC coatings [30]. It is suggested that rough surfaces in comparison with smooth surfaces improve cell attachment due to the formation of specific surface-cell contacts by increasing the expression of different integrins subunits [40,41]. Although, diverse authors have reported that surface roughness values higher than 0.5 µm are desirable to ensure the maximum attachment and proliferation of cells, large rough surfaces also stimulate more anti-inflammatory responses because the activation of M2 macrophages and the subsequent release of anti-inflammatory cytokines [42]. The PEDOT:κC:Dx surface roughness value and the lack of their significative variation during the delivery of dexamethasone may indicate the reliability of electroactive composite for cell culture studies, since no additional mechanism may be seemed due to the topography changes.

**Figure 3:**
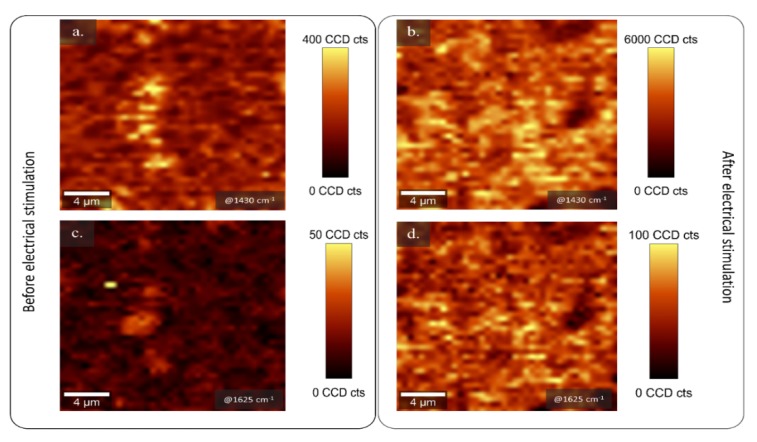
2D confocal Raman map of the 1430 cm^−1^ band (**a**) before release process and (**b**) after 160 release cycles. Raman mapping of the 1625 cm^−1^ band intensity (**c**) before release process and (**d**) after 160 release cycles at 0.5 µm depth inside the conductive layer. The yellow areas are related to the presence of PEDOT and κC/Dx, respectively.

The qualitative composition of the conductive film was determined using confocal µ-Raman spectroscopy before (Figure 3a,c) and after (Figure 3b,d) 160 sweeps of electrical stimulation in a 4 µm^2^ area and 5 µm depth inside the composite. The analysis was performed in order to determine the presence of PEDOT, dexamethasone, and κ-carrageenan inside the electroactive composite. The signal was obtained and plotted in a 2D image that allows the association of the signal (counts) to the presence of the corresponding functional groups for each component.

PEDOT shows a strong signal in the spectral range of 1421–1442 cm^−1^, associated to the thiophene symmetric C_α_ = C_β_ stretching [2,30,43] and its oxidation state. The corresponding signal was obtained from the composite before and after 160 cycles of electrical stimulation (Figure S2) and it was mapped at 1430 ± 25 cm^−1^ (Figure 3a,b), where bright yellow dots corresponded to presence of PEDOT. A homogeneous distribution of the conductive polymer was detected in both samples.

Additionally, a relative intense band at 1625 ± 30 cm^−1^ was detected, corroborating the qualitative existence of Dx and κC in the conductive film (Figure 3c,d). This signal, in the 2D, is distributed through the conductive matrix. The result is similar to previous studies [13,26], which reported the characteristic spectral signals of dexamethasone in the ranges of 3200–3500 cm^−1^, 2850–3000 cm^−1^, and near to 1650 cm^−1^, as is verified in Figure S3, corresponding to hydroxyl, methyl, and carbonyl groups, respectively. Dexamethasone and κC act as doping agents, so there is a consistent association of the respective signal for both molecules and the PEDOT band. The identification of the band at 1625 cm^−1^ overlapping with PEDOT signal, confirmed the presence of the doping agent before and even after electrochemical stimulation, as is shown in Figure S2a,b, respectively. Adding κC in the formulation provides a proper doping agent during the release of the Dx, reducing the degradation by overoxidation and eventually delamination as is shown in Figure S4 [30].

### 2.3. Dexamethasone Release Experiments from the PEDOT:κC:Dx Coating

Drug loading into the conducting polymers films is based on the fact that these kinds of polymers are electrically oxidized during the polymerization processes, generating charge carriers [9,44,45]. The doping agent (e.g., Dx and κC) is incorporated to the oxidized polymer [46] to maintain charge neutrality. In this work, dexamethasone 21 phosphate and κC are used as doping agents, the presence of sulfate and phosphate groups imparts negative charges in the polysaccharide and the drug, respectively.

**Figure 4:**
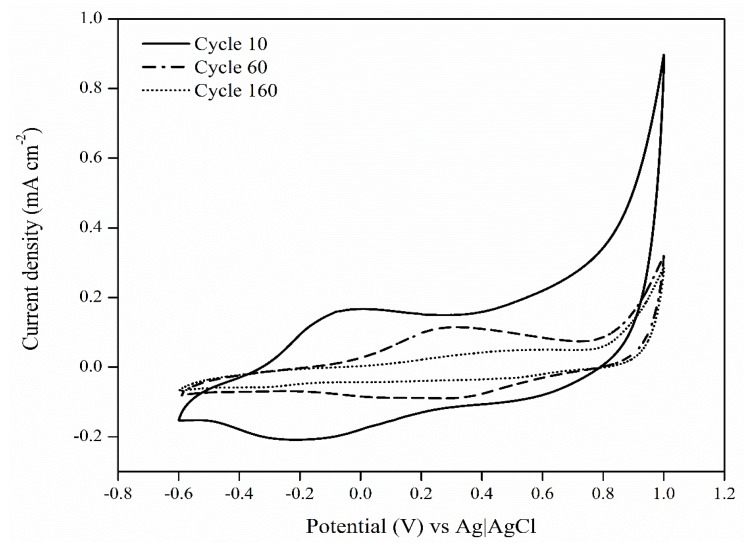
Cyclic voltammograms for the PEDOT:κC:Dx recorded at 25 mV·s^−1^ after 10, 60, and 160 cycles of electrical stimulation in ammonium acetate 0.10 M.

The electrochemical controlled release studies from PEDOT:κC:Dx coating were performed within a potential range of −600 to 1000 mV to evaluate intrinsic redox processes of the film [13,35,45]. Figure 4 shows the characteristic oxidation and reduction potential signal ranges at 0 to 500 mV and −100 to −400 mV, respectively, after a different number of voltammetry scans. According to some authors, the voltammetric behavior of dexamethasone shows a reduction signal at the potential of −350 mV [13,45], which indicates the release of the drug from a stimulated electrode. The corresponding CV signals are shown in Figure 4, this signal gradually decreased according to the sweep number, disappearing completely after 160 cycles of electrical stimulation. Electrochemical reduction of a conducting polymer results in the migration of small doping molecules from the conducting composite to maintain the electro neutrality of the matrix [44,46]. Thus, the application of alternating positive and negative potentials during cyclic voltammetry analysis caused the release of the Dx from the PEDOT coating.

Spontaneous release of the dopant from the PEDOT structure is an instant process, but the Dx release is slow, since it is driven by diffusion from the inner film to the surface. κC is a large molecule, this type of dopant is more attached into the polymer coating and it is not leached out during the electrical stimulation, granting to the polymer greater electrochemical stability [13,46,47], as confirmed by Raman spectroscopy.

**Figure 5:**
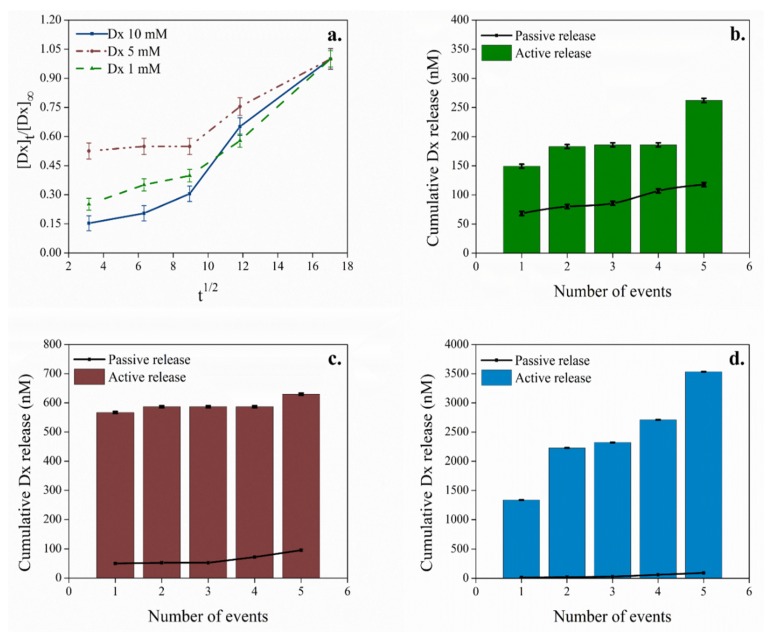
(**a**) The passive release profile of Dx as a function of square root of time, over 300 min from unstimulated electrodes. The active electrically controlled delivery process by stimulation events (columns) compared to the passive release profile (line) using: (**b**) 1 mM, (**c**) 5 mM, and (**d**) 10 mM of Dx in the initial formulation.

The release profile of the Dx was investigated under passive conditions (unstimulated) and active electrically stimulation using an ammonium acetate 0.10 M solution as supporting electrolyte. The surface area of the electrode is associated with promoting larger amounts of passive drug release according to the second Fick’s law of diffusion [48,49], yet, in our case, the electrode surface and total area are maintained virtually constant. The quantification of Dx from the PEDOT:κC:Dx modified electrodes was achieved using HR-mass spectrometry (Figure 5).

The active release profile was performed with a total of 76 CV sweeps in five release events, taking around 300 min to be completed. Accordingly, the passive release profile from unstimulated electrodes were evaluated over the same period of 300 min.

Figure 5a shows the passive release profile of Dx as a function of square root of time according to the Higuchi model for the drug release from a polymer film [27,50], where pure Fickian diffusion is the dominant phenomena [48]. The low diffusion value, in the beginning of the process, may depend on the slow penetration of supportive electrolyte into the polymeric film [49]. The pattern changed after 80 min and a higher diffusion value reflects the diffusivity of the passive Dx release process. The three systems (1 mM, 5 mM, and 10 mM) showed analogous Fickian diffusion behavior.

On the other hand, Figure 5b–d showed a remarkable dependency of the released Dx concentration during the electrical stimulated events (bars) compared to a passive unstimulated electrode (line). Some authors have studied controlled drug release systems using conductive polymers such as polypyrrole and PEDOT, where the anionic molecule is used as doping agent and their subsequent release is mainly determined via diffusion [11,13,44,45,51]. Nevertheless, for a controllable release system, it is desirable to have a high active release and low diffusion relationship [11,12], as shown by our system (see Figure 5). For instance, the initial concentration of 10 mM released in the passive process ca. 2% of the delivered Dx in stimulated process. This is probably associated with the use of κC as second doping in the matrix, which grants the film stability and integrity during stimulation cycles [30,46].

The therapeutic dosages of Dx in mesenchymal stem cell cultures are effective at levels of 100–1000 nM to promote their differentiation to osteoblast or in order to be used during anti-inflammatory treatment [52,53,54]. In this work, the accumulative concentration of the released Dx using 1 mM and 5 mM initial formulations (Figure 5b,c) were 300 nM (0.66 µg·cm^−2^) and 600 nM (1.60 µg·cm^−2^), respectively. Even though, these values are at therapeutically relevant levels, they are in part determined by the Dx amount release via diffusion.

Instead, when 10 mM of the drug was poured in the initial formulation, a total of 3700 nM (8.89 µg·cm^−2^) of cumulative Dx was detected. This concentration range far in excess of the quantity of dexamethasone released from similar systems using an identical initial concentration of the drug for the coating preparation, for which values are even lower than 5.03 µg·cm^−2^ [11,12,51]. Such concentrations surpass the amount of the drug needed in cell cultures and it is not recommended to apply in biological systems. Nonetheless, using a specific electrochemical stimulation profile may be allowed to provide an adequate quantity of the drug for different biological applications.

## 3. Materials and Methods

### 3.1. Materials

Monomer 3,4-ethylenedioxythiophene (EDOT, 97.0% purity), κ-carrageenan (κC, ACS reagent), potassium chloride (KCl, >99.0% purity), dexamethasone 21-phosphate disodium salt (Dx, 98.0% purity), ammonium acetate (NH_4_CH_3_CO_2_, 98.0% purity), ultrapure water MS quality, and MS methanol were purchased from Sigma Aldrich (San José, Costa Rica). All chemical reagents were used without further purification.

### 3.2. Synthesis and Preparation of the Modified PEDOT:κC:Dx Electrode

Electrodes (20.49 ± 0.02 mm^2^) were fabricated by the deposition of gold on a polyimide substrate (see Figure S4) and they were passivated using a shadow mask to leave a specific exposed area to the electrode [55]. Prior to the polymer deposition, all electrodes were electrochemically cleaned applying cyclic voltammetry (CV) sweeps from a range of −600 to 900 mV with 100 mV·s^−1^ scan rate, in KCl 0.2 M [56], using an Autolab Potentiostat supplied by Metrohm (PGSTAT-302N, AUTOLAB, Utrecht, The Netherlands).

The surfactant dispersion was prepared according to a previous work [30], briefly: κC (0.2% *w*/*v*) and KCl (0.2 M) were added to deionized water previously heated at 50 °C. The samples were sonicated using 140 Joules in a Sonifier QSonica (Q700, Ultrasonic Corporation, Danbury, CT, USA), before and after adding the monomer EDOT (10 mM) and Dx at three different concentrations: 1 mM, 5 mM, and 10 mM.

The solution was electropolymerized on the electrode surface using galvanostatic conditions in the Autolab Potentiostat. The gold electrode (see Figure S4) is used as working electrode, platinum as counter electrode, and Ag|AgCl (KCl 3.0 M) works as reference electrode. The electrical polymerization was carried out with a constant current of 102.45 microamperes (current density: 0.5 mA·cm^−2^) using a potential limit of 1400 mV during 360 s (ca. 180 mC·cm^−2^ of charge density). Following the PEDOT:κC:Dx deposition, the electrodes were intensively rinsed with deionized water and stored at 4 °C before their use.

### 3.3. Evaluation of the Stability and Size of the Dispersion Systems

The characterization of the particle size and ζ-potential was performed using six dispersions, prepared in deionized water, namely: (1) κC 0.2% *w*/*v*; (2) Dx 10 mM; (3) EDOT 10 mM:κC 0.2% *w*/*v*; (4) EDOT 10 mM:Dx 10 mM; (5) κC 0.2% *w*/*v*:Dx 10 mM; and (6) EDOT 10 mM:κC 0.2% *w*/*v*:Dx 10 mM. Measurements were performed in a Zetasizer instrument (Nano ZS, Malvern Panalytical Ltd., Worcestershire, UK) at 25 °C and 173° angle. All the measurements were done by triplicate. Finally, dispersions were sonicated using a high-power ultrasonic bath (Bransonic^®^, Merck corporation, San José, Costa Rica) for 6 min to promote their homogenization. Two more formulations of EDOT:κC:Dx were prepared to reach lower dexamethasone concentrations into the conductive layer.

### 3.4. Analysis of the Topography and Composition of PEDOT:κC:Dx Coating by Profilometry and µ-Raman Spectroscopy Methods

The electrode topography was studied by profilometry analysis (Bruker, model: Dektak TX Advance, AZ, USA) and the arithmetical mean roughness of the surface (Sa) was calculated to describe the topography of the materials by using a 2 μm tip radius and a force of 1 mg in a 300 × 300 μm^2^ and a scan area rate of 2.5 μm·s^−1^.

Raman spectroscopy analysis was carried out using a confocal µ-Raman microscope (Alpha300 R WITec, GmbH, Ulm, Germany) with a 532nm excitation laser, exposure time of 0.5 s, and 105 accumulations. The Raman stack scan was obtained using an integration time of 4 s in 4 µm^2^ of area, 200 measurements per line were recorded for a total of 20 lines in each stack. Oversampling was used to improve the image quality, which was done in case of the cross-sectional scan. The scan depth was fixed at 5 µm and a total of 10 stack scans were achieved. The intensity of the relative wavenumber at 1435 cm^−1^ and 1625 cm^−1^ were extracted from each acquired spectrum, corresponding to PEDOT [2] and Dx/κC [44,57], respectively and plotted as 2D image. The intensity counts are related to the presence of the functional group and it is presented as bright yellow areas.

### 3.5. Dexamethasone Release Experiments from the PEDOT:κC:Dx Film

The Dx release from the modified electrode was carried out in a continuous flow cell using cyclic voltammetry (CV) sweeps with a three electrodes system (PEDOT:κC:Dx, Ag|AgCl and a gold film as working, reference, and counter electrodes, respectively). The active release of the drug was performed in 1 mL of fresh ammonium acetate solution (0.10 M) pH 7.2 [58], by scanning of CV from −600 to 1000 mV with a 25 mV·s^−1^ scan rate, over a period of 300 min (5 samples total) at room temperature.

The second release event, without electrical stimulation, was performed in order to analyze and to quantify the passive drug release process. For the experiment, 1.0 mL of 0.10 M ammonium acetate was injected through the cell containing the electrodes, a total of five samples were collected during 300 min of analysis.

Dexamethasone phosphate concentration, in the samples for the active and passive release events, was determined using a Xevo G2-XS quadrupole time of flight (Q-tof) mass spectrometer (Waters Corporation, Wilmslow, UK) coupled with an Acquity UPLC H-Class. For the analysis, a 10-µL injection of the sample was separated with an Acquity UPLC^®^ C18 column (2.1 mm × 50.0 mm). The mobile phase consisted of a solution of water:formic acid 0.05% *v*/*v* and methanol:formic acid 0.05% *v*/*v* and they were supplied under not isocratic conditions with a constant flow of 0.3 mL·min^−1^ (Table S1).

The mass spectrometer was configured according to the parameters in a previous work [59], with the modifications shown in supplementary information S1. Quantification was carried out using Multiple Reaction Monitoring (MRM) acquisition method with the optimized transition of 471.1584 *m/z* for the precursor ion and 78.9585 *m/z* for the product ion, with a collision energy of 35 eV. Concentration in each sample was calculated using the Software MassLynx™ (V4.1, Waters Corporation, Wilmslow, UK) and an external calibration curve between 0.5 ppb to 5000 ppb of dexamethasone phosphate (R^2^ = 0.9965).

## 4. Conclusions

We have successfully delivered therapeutic doses of dexamethasone by an electroactive controlled system, adjusting the initial formulation and the electrical stimulated events. Moreover, using κ-carrageenan as dispersant during the polymerization and as a doping agent in the composite, we avoided delamination and changes in the film roughness. The chemical composition inside the conductive film was confirmed by 2D Raman and electrochemical signal in the cyclic voltammetry analysis. Concentrations of dexamethasone in the range of 100 to 1000 nM were obtained using a lower amount of dexamethasone in the initial formulation. Those concentrations are recommended to induce differentiation in mesenchymal cell cultures and in anti-inflammatory responses. Therefore, an adequate formulation along with a proper active electrochemical stimulation profile allowed the delivery of therapeutic doses of charged molecules without significant changes in our film roughness. Our approach may be useful in the development of diverse strategies and implant systems in the regenerative medicine field.

## Figures and Tables

**Table 1:** ζ-potential values of dispersions used in the fixation of the drug on the electrode.

System	ζ-potential (mV)	SD (mV)
Dx	−69.40	1.14
κC	−43.30	3.31
κC:Dx	−42.63	1.67
EDOT:Dx	−70.83	1.09
κC:EDOT	−48.46	1.70
EDOT:κC:Dx	−48.70	1.21

## Supplementary Materials

The following is available online, Figure S1: Galvanostatic curve of the electro-polymerization process from an EDOT:κC:Dx dispersion onto a bare gold electrode. Figure S2. Raman spectra of the PEDOT:κC:Dx coating (a.) before dexamethasone release process (Inset: PEDOT:κC:Dx electrode surface) and (b.) after 160 release cycles (Inset: PEDOT:κC:Dx electrode surface). Figure S3. µ-Raman spectral measurement of the dexamethasone 21-phosphate disodium salt. Figure S4. Deposited PEDOT:κC:Dx electrode after 160 cycles of electrical stimulation (left) and gold electrode without passivation as reference (right). Table S1: Gradient elution method for the mobile phase using during dexamethasone analysis. Solvents were water: 0.05% formic acid (A) and methanol: 0.05% formic acid (B). and S1: Configuration of the mass spectrometer during dexamethasone quantification.
